# Hammer Nose

**Published:** 1889-11-02

**Authors:** 


### HAMMER NOSE.

On "Rhinophyma, or the Hammer Nose," is the title of a paper by Dr. Balmanno Squire, surgeon to the British Hospital for Diseases of the Skin, and which has been published in a separate form. The article is so exhaustive of the subject that we can scarcely do more than touch on some of the salient points. "Hammer nose," or as sometimes termed "bottle nose," is a condition which derives its importance from the disfigurement of the nose which it occasions. It chiefly affects the male sex, attains its maximum development in elderly men, and is commonly supposed to be the result of over-indulgence in good living and in alcoholic drinks. The pathological condition appears to be a pseudo-hypertrophy of the skin. The new formation consists principally of embryonic connective tissue, which later on undergoes development into mature connective tissue. With this thickening of the skin is associated dilatation of the smaller bloodvessels of the skin, and of the sebaceous follicles. Of the tumour, there are usually three lobes, two lateral and one in front.

The view Dr. Squire takes of rhinophyma is that it is simply one of the degenerations of advanced age, and that the affection is often hereditary. In every country, artists and actors are in the habit of representing drinkers as affected with rhinophyma. But, as a matter of fact, the "jolly nose" of drinkers is not rhinophyma, but *rosacea*. Although alcoholised persons are not exempt from rhinophyma, they are not more subject to it than other people. The treatment of rhinophyma is in most instances a surgical matter. In the earlier stages it is possible to reduce the swelling by pressure (so as to express sebaceous accumulations), and by the application of red iodide of mercury ointment (15 grains to the ounce). When the disease has attained considerable development pedunculated excrescences may be snipped off. In other cases a wedge-shaped piece of skin may be removed. Complete ablation may also be necessary; and the author gives the drawing of an instrument he uses for this purpose. But, as a rule, rhinophyma gives no pain, and most elderly men would rather bear the ills they have than submit to the surgical knife.

